# Inflammation produced by senescent osteocytes mediates age-related bone loss

**DOI:** 10.3389/fimmu.2023.1114006

**Published:** 2023-02-06

**Authors:** Zixuan Wang, Xiaofei Zhang, Xing Cheng, Tianxing Ren, Weihua Xu, Jin Li, Hui Wang, Jinxiang Zhang

**Affiliations:** ^1^ Department of Emergency Surgery, Union Hospital, Tongji Medical College, Huazhong University of Science and Technology, Wuhan, China; ^2^ Center for Translational Medicine, Union Hospital, Tongji Medical College, Huazhong University of Science and Technology, Wuhan, China; ^3^ Health Care Management Center, Union Hospital, Tongji Medical College, Huazhong University of Science and Technology, Wuhan, China; ^4^ Department of Orthopedics, Union Hospital, Tongji Medical College, Huazhong University of Science and Technology, Wuhan, China; ^5^ Department of Medical Genetics, Basic School of Tongji Medical College, Huazhong University of Science and Technology, Wuhan, China

**Keywords:** age-related bone loss, senescence, inflammation, proteomics, osteocyte, rapamycin

## Abstract

**Purpose:**

The molecular mechanisms of age-related bone loss are unclear and without valid drugs yet. The aims of this study were to explore the molecular changes that occur in bone tissue during age-related bone loss, to further clarify the changes in function, and to predict potential therapeutic drugs.

**Methods:**

We collected bone tissues from children, middle-aged individuals, and elderly people for protein sequencing and compared the three groups of proteins pairwise, and the differentially expressed proteins (DEPs) in each group were analyzed by Gene Ontology (GO) and Kyoto Encyclopedia of Genes and Genomes (KEGG). K-means cluster analysis was then used to screen out proteins that continuously increased/decreased with age. Canonical signaling pathways that were activated or inhibited in bone tissue along with increasing age were identified by Ingenuity Pathway Analysis (IPA). Prediction of potential drugs was performed using the Connectivity Map (CMap). Finally, DEPs from sequencing were verified by Western blot, and the drug treatment effect was verified by quantitative real-time PCR.

**Results:**

The GO and KEGG analyses show that the DEPs were associated with inflammation and bone formation with aging, and the IPA analysis shows that pathways such as IL-8 signaling and acute-phase response signaling were activated, while glycolysis I and EIF2 signaling were inhibited. A total of nine potential drugs were predicted, with rapamycin ranking the highest. In cellular experiments, rapamycin reduced the senescence phenotype produced by the H_2_O_2_-stimulated osteocyte-like cell MLO-Y4.

**Conclusion:**

With age, inflammatory pathways are activated in bone tissue, and signals that promote bone formation are inhibited. This study contributes to the understanding of the molecular changes that occur in bone tissue during age-related bone loss and provides evidence that rapamycin is a drug of potential clinical value for this disease. The therapeutic effects of the drug are to be further studied in animals.

## Introduction

Bone is a dynamic organ in which bone formation mediated by osteoblasts balances against bone resorption mediated by osteoclasts to maintain bone homeostasis (1). With age, this balance gradually tilts toward bone resorption, leading to bone loss and osteoporosis (2, 3). The most important complication of osteoporosis is fracture (4), which leads to increased mortality and makes a significant impact on the health and quality of life of patients (5). As the population ages, the incidence of fractures due to osteoporosis is also increasing, which is a major health problem (6).

Bone senescence is a highly complicated process, which results from the interplaying of systemic and local factors with a variety of bone-related cells, including osteocytes, osteoblasts, osteoclasts, bone-marrow-derived mesenchymal stem cells (BMSCs), and bone-marrow-derived macrophages (BMDMs) in response to various intracellular and extracellular stimuli, such as oxidative stress, genetic damage, and the altered responses of bone cells to various biological signals and to mechanical loading (7). During bone aging, senescent osteocytes and myeloid cells are the main sources of senescence-associated secretory phenotype (SASP) in the bone microenvironment, and the expression levels of SASP components including p53, p21, and p27 were significantly elevated (8). SASP is the most important feature of senescent cells and is a conserved cellular response that manifests as a low-grade chronic inflammatory state that emerges with age (9). The pro-inflammatory phenotype of SASP is mediated by NF-κB cascade amplification signals (10).

A hallmark of the aging process is a progressive increase of chronic inflammation, which was originally called “inflamm-aging” (11). Although restricted inflammation is beneficial for bone repair, systemic chronic inflammation yielding excessive proinflammatory cytokines such as IL-1, IL-6, and TNF is detrimental to bone formation and fracture healing (12). Macrophages were considered as the primary player in mediating the inflammatory responses (13). However, several studies indicated that aged macrophages are less responsive to IFNγ or LPS by secreting the lower levels of inflammatory cytokines (14, 15). Osteocytes, accounting for over 90% of the bone cells, can transmit signals to each other by forming a network of tubules through axons (16). Current studies have shown that bone tissue expression of pro-inflammatory factors is elevated in mice with osteoporosis, such as TNF-α (17), IL-6 (18), and IL-1 (19). Nevertheless, the cells mainly mediating aging-associated inflammatory responses are unclear.

Proteins are the most important functional executor in a living organism. Proteomics based on label-free liquid chromatography-mass spectrometry (LC-MS/MS) routinely quantifies thousands of proteins across multiple samples in a single run, the following annotation providing an important path for the study of disease pathology and the discovery of therapeutic targets. Several groups have performed a proteomics approach to explore the pathology of bone-related diseases, including osteoporosis (20, 21), osteosarcoma (22, 23), osteoarthritis (24), and bone fracture (25). Most of the proteomics studies used cultured cell samples, including BMSCs (26–29), osteoblasts (30, 31), and osteoclasts (32–34). However, the proteomic alteration of cultured cells in response to a certain stimulus cannot simulate the actual situation of bone tissues *in vivo*. Moreover, previous proteomics studies on human bone tissues are scarce, and the overall research in bone primarily focused on genomics and transcriptomics (35). It might result from the lack of access to obtain in clinics and the costs. Also, postmenopausal osteoporosis cannot be equated with age-related bone loss. In addition to all the above restraints, proteomics analysis about bone aging was limited so far.

In the present study, the bone specimens from children, middle-aged patients, and older individuals were subjected to proteomics analysis by LC-MS/MS. The differentially expressed proteins (DEPs) from the pairwise comparison or from three groups continuously up- or downregulated with age were annotated. We also compared and investigated the possibility of osteocytes as the main cells producing the inflammatory-associated DEPs or signaling pathways during bone aging. In addition, rapamycin was predicted as an inhibitor of bone aging. Finally, we confirmed the reliability of our proteomics results and the effect of rapamycin on the expression of the inflammatory or SASP marker genes. Our study will advance a better understanding of the molecular mechanisms of bone aging.

## Methods

### Collection of human samples

The project was approved by the Ethics Committee of the Union Hospital of Tongji Medical College, Huazhong University of Science and Technology (Ethics No. 2020-S001). The procedure was according to approved guidelines. Human bone samples were collected from patients undergoing surgical treatment in the orthopedic surgery department at Union Hospital. These bone samples would usually have been discarded as part of joint replacement surgery or associated surgery. The study included 33 subjects, with 11 samples from 2 to 12 years old, 11 samples from 41 to 54 years old, and 11 samples from 69 to 88 years old. Subjects who had tumors or systemic diseases, were immunologic, were treated with steroids or hormones, or had other factors that might affect bone metabolism were excluded. In order to avoid the influence of bone-related diseases on the local bone microenvironment, we sampled the site as far away from the lesion as possible. When the tissue is collected, it is washed using saline to remove blood from the surface, then stored in liquid nitrogen. The collected bone tissue does not contain bone marrow or cartilage tissue, its cellular component is mainly osteocytes, and other components include mineral salts and various proteins (collagen and non-collagen). The basic information of the 33 individuals and the anatomical sites from which the samples were collected are included in **Supplementary Table 1**.

### Label-free quantitative proteomics analysis

The bone tissue was fully ground to a powder by adding liquid nitrogen, and each sample was lysed by adding 4 times the volume of powder lysis solution (1% SDS, 1% protease inhibitor), sonicated at 4°C, and centrifuged at 12,000*g* for 10 min. The supernatant was transferred to a new centrifuge tube for protein concentration determination using a BCA kit. Trypsin was added and enzymatically cleaved into peptide fragments. The peptides were dissolved with liquid chromatography mobile phase A and separated using EASY-nLC 1200 UHPLC system and then injected into an NSI ion source for ionization and then into a mass spectrometer (Q Exactive™ HF-X) for analysis. The data acquisition mode was performed using a data-dependent scanning (DDA) program.

### Functional enrichment analysis

Proteins in the three groups were compared with each other, and proteins with *p*-value <0.05 and fold change >1.5 or <1/1.5 determined by Student’s *t*-test were defined as differentially expressed proteins (DEPs). Pearson’s correlation coefficient was used to detect correlations between groups of samples, visualized by TBtools (36). The Kyoto Encyclopedia of Genes and Genomes (KEGG) and Gene Ontology (GO) functional enrichment analyses were performed on the DAVID database (https://david.ncifcrf.gov/), and the parameter settings are all default values. The results of the GO analysis were plotted using GraphPad Prism 8.0, and the results of KEGG analysis were visualized using an online platform (http://www.bioinformatics.com.cn). To analyze protein temporal changes with age, the DEPs were analyzed by the k-means clustering algorithm and then visualized by an online platform (http://www.bioinformatics.com.cn).

### Ingenuity pathway analysis

Ingenuity Pathway Analysis (IPA) was used to predict the activation or inhibition state of the canonical pathway (37), and it was analyzed based on the reported literature. The lists of DEPs were uploaded to the IPA software (QIAGEN). The “core analysis” of DEPs was first performed in the software, and the results can be obtained for the canonical signaling pathways and upstream regulatory molecules. In addition, a “comparative analysis” can be performed for the pairwise comparison groups. Utilizing the software, predictions are scored by *z*-score: when the *z*-score is greater than or equal to 2, predictions are activated, and when the *z*-score is less than or equal to −2, predictions are suppressed.

### Connectivity map analysis

To explore potential drugs by Connectivity Map (CMap) analysis (https://clue.io/query), the dataset allows for drug prediction based on gene changes. So, we predicted potential therapeutic drugs by targeting proteins that change when age-related bone loss occurs. The database scores all predicted drugs from −100 to 100. All drugs predicted were selected for the generation of a heatmap according to the scores. A score of 100 means that the drug produces exactly the same perturbation as the change in the input gene, while −100 means that the drug produces a perturbation exactly opposite to the change in the input gene. When screening for therapeutic drugs, drugs with changes opposite to the DEPs and scores less than −90 are considered meaningful.

### GSEA

Gene set enrichment analysis (GSEA) was performed using the pre-ranked method in GSEA Java (http://software.broadinstitute.org/gsea/msigdb), and genes from GSE141595 were used for the analysis (8). For our study, we used all the C5 collection and interesting signaling pathways related to inflammation for GSEA. The minimum and maximum numbers for the selection of gene sets from the collection were 10 and 500 genes, respectively.

### Animals

All experimental procedures involving animals were approved by the Animal Care and Use Committee of Wuhan Union Hospital (Ethic No.3047). Three of each of the 6-week-old (young) and 18-month-old (old) C57BL/6J mice were bought from Beijing Vital River Laboratory Animal Technology (Beijing, China). Mice were anesthetized with sodium pentobarbital (60 mg/kg intraperitoneally) and subsequently executed by cervical dislocation followed by immersion in 75% alcohol for 5 min. The mouse skin and muscle were scissored to separate the mouse tibia and femur. The bone marrow cavity of the mice was opened in a sterile operating table and then flushed with PBS to remove the bone marrow, leaving the bony part. Bones from each mouse were mixed and placed in liquid nitrogen and then ground with a mortar and pestle. Bone pieces were lysed in 1*RIPA buffer (Beyotime, China) with proteinase inhibitor cocktail (Beyotime, China) for 15 min at 4°C. Bone debris was removed after centrifugation at 3,000 rpm for 5 min at 4°C. Bone samples were stored at −80°C for the subsequent experiments.

### Cell culture

MLO-Y4 cells were utilized as osteocytes in our research which were bought from iCell Bioscience (China). They were cultured in 12-well plates in α-MEM supplemented with 10% FBS and 1% PS. Mild concentrations of H_2_O_2_ at 400 μM for 12 h were utilized to construct an induced senescent phenotype (38, 39), and then the phenotype was treated with different concentrations of rapamycin for 24 h.

### Quantitative real-time PCR

The total RNA of MLO-Y4 cells was extracted by TRIzol (Biosharp), and cDNA was reverse-transcribed using HiScript 1st Strand cDNA Synthesis Kit (Vazyme) and real-time PCR using SYBR qPCR Mix (Vazyme). The primer sequences were as follows: β-actin (mouse): 5′-CATTGCTGACAGGATGCAGAAGG-3′ (forward) and 5′-TGCTGGAAGGTGGACAGTGAGG-3′ (reverse); IL-6 (mouse): 5′-TACCACTTCACAAGTCGGAGGC-3′ (forward) and 5′-CTGCAAGTGCATCATCGTTGTTC-3′ (reverse); P53 (mouse): 5′-CCTCAGCATCTTATCCGAGTGG-3′ (forward) and 5′-TGGATGGTGGTACAGTCAGAGC-3′ (reverse); P21 (mouse): 5′-TCGCTGTCTTGCACTCTGGTGT-3′ (forward) and 5′-CCAATCTGCGCTTGGAGTGATAG-3′ (reverse); P27 (mouse): 5′-AGCAGTGTCCAGGGATGAGGAA-3′ (forward) and 5′-TTCTTGGGCGTCTGCTCCACAG-3′ (reverse); and Opg (mouse): 5′-CGGAAACAGAGAAGCCACGCAA-3′ (forward) and 5′-CTGTCCACCAAAACACTCAGCC-3′ (reverse).

### Western blot analysis

The human and mouse bone protein lysates were loaded into 10% SDS-PAGE gels, and the gels were cut into two parts. They were transferred into a 0.45-μm polyvinylidene difluoride membrane (Millipore) and separated. The large molecule protein CSPG4 (A3592, ABclonal) was processed at 300 mA for 3 h at 4°C with 10% methanol, and ITGA2B (A5680, ABclonal), tubulin (GB11017, Servicebio), and β-actin (GB11001, Servicebio) were processed at 300 mA for 1.5 h at 4°C with 20% methanol. The intensity of the protein was analyzed with ImageJ software.

### Statistical analysis

Student’s *t*-test was the statistical method used to compare protein sequencing results. GraphPad Prism 8.0 was used to perform one-way ANOVA with Bonferroni correction for comparisons among more than two groups in the cellular experiments. Significance was determined at *p <*0.05. All experiments were performed at least in triplicate and in three independent experiments.

## Results

### Characterization of proteomics of human bone tissues at different ages

**Figure 1:**
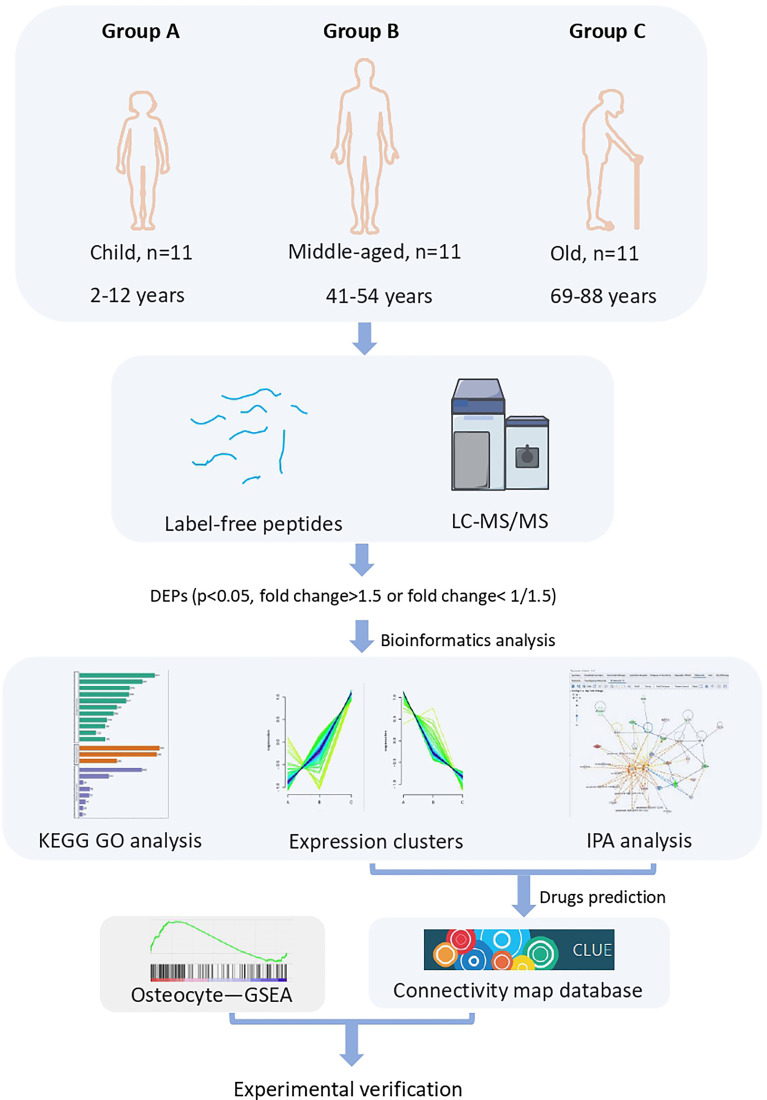
Flow diagram of label-free LC-MS/MS proteomics.

To identify the key proteins/pathways and candidate biomarkers during bone aging, we performed label-free LC-MS/MS proteomic sequencing on bone tissues from the three cohorts: children (group A), middle-aged individuals (group B), and older individuals (group C). As shown in **Figure 1**, the DEPs (*p* < 0.05, fold change > 1.5 or fold change < 0.667) were subjected to further bioinformatic analysis, including GO analysis, KEGG analysis, and IPA analysis. The potential drugs to treat bone aging were also predicted based on the DEPs, and we also verified the expression of several key DEPs and the effect of the predicted drugs on bone cell senescence (**Figure 1**).

**Figure 2:**
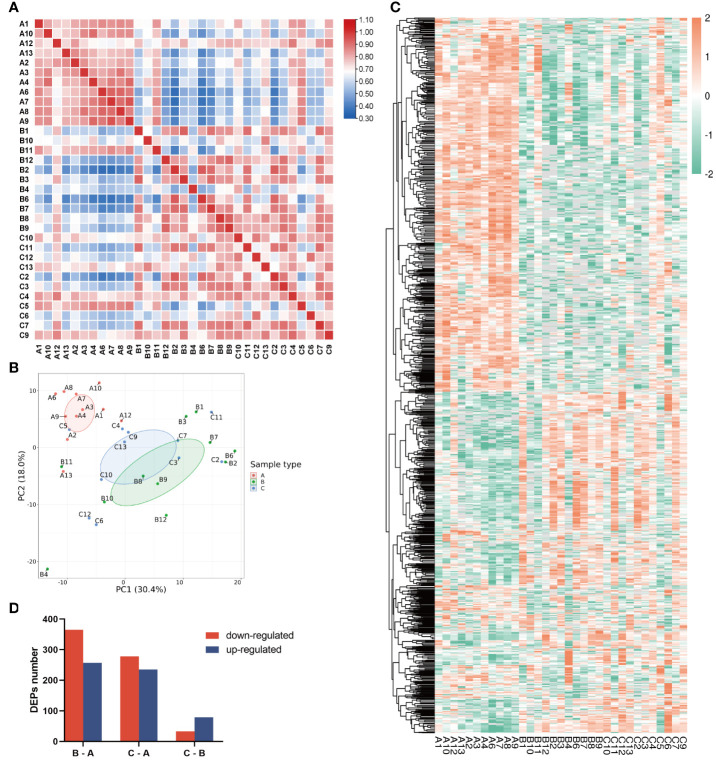
Characterization of proteomic of human bone tissue at different ages. **(A)** Pearson’s correlation matrix of 33 samples. The color of the square represents the magnitude of the correlation: blue represents a small correlation coefficient, while red represents a large correlation coefficient as the color bar shows. **(B)** PCA plot of the three groups. Group A was distinct from groups B and C. **(C)** Heatmap of all protein expression in the three groups. **(D)** The numbers of differentially expressed proteins (DEPs) in the three pairwise-compared groups. The red bar indicates the downregulated proteins, and the blue bar indicates the upregulated proteins. In the chart, group A refers to the children, group B refers to the middle-aged individuals, and group C refers to the older individuals.

As shown by Pearson’s correlation analysis (**Figure 2A**) and principal component analysis (PCA, **Figure 2B**), the within-group variation is relatively low in the children group, whereas the variations are high in both the middle-aged group and the older group, implying large individual differences after bone maturation. Moreover, the children group was significantly different from the other two groups. Accordingly, there is a great difference in protein profiling between the children group and the other two groups which had some overlapped individuals (**Figure 2B**). The heatmap of DEPs also shows more DEPs between the children group and the other two groups (**Figure 2C**). As shown in **Figure 2D**, the total number of DEPs when comparing the middle-aged and children groups (B–A) is 622, of which 365 were downregulated and 257 were upregulated. There are 513 DEPs with 278 downregulated and 235 upregulated in the bone tissues from the older group compared with the children group (C–A). Only a small number of DEPs (112) were found between the older group and the middle-aged group (C–B). All data indicated that the proteins in bone tissues were differentially expressed with aging.

### Analysis of the DEPs from the pairwise comparison

**Figure 3:**
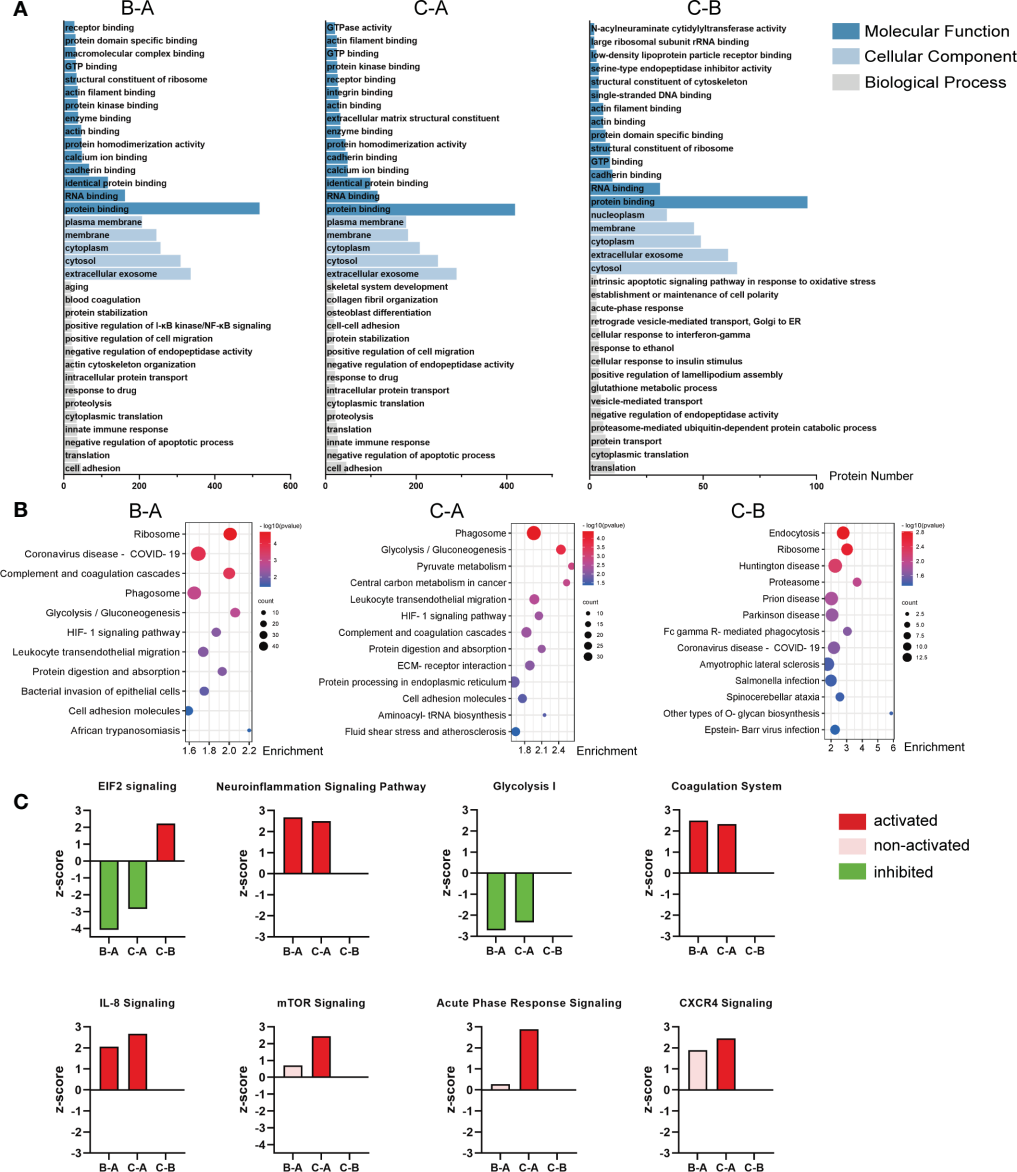
Analysis of the DEPs from the pairwise comparison. **(A)** Representative GO enrichment in the three pairwise comparison groups; the horizontal axis indicates the number of enriched genes. **(B)** Bubble plot of KEGG enrichment analysis of the three comparison groups; the color of the bubble represents the enriched *p*-value, and the size of the bubble represents the number of enriched genes. **(C)** Activation or inhibition of several canonical signaling pathway in the three comparison groups. *Z*-score >2 means the pathway is activated, indicated in red, while *z*-score <−2 means the pathway is inhibited, indicated in green; the pink bar means non-activated pathway; and the missing values in the C–B groups mean no valid prediction. In the chart, B–A refers to middle-aged individuals compared with children, C–A refers to older patients compared with the middle-aged individuals, and C–B refers to older patients compared with middle-aged individuals.

The DEPs from the pairwise comparison between middle-aged individuals and children (B–A), older individuals and children (C–A), or older and middle-aged individuals (C–B), respectively, were annotated to GO and KEGG analyses. **Figure 3A** shows the results of GO analysis for the three paired comparison groups, and the top 15 molecular functions, the top 5 cellular components, and the top 15 biological processes were listed. The complete GO analysis data are listed in **Supplementary Table 2**. Notably, in the B–A groups, biological processes were enriched in aging, blood coagulation, positive regulation of I-κB kinase/NF-κB signaling, and innate immune response (**Figure 3A**, left). In the C–A groups, biological processes were enriched in skeletal system development, collagen fibril organization, osteoblast differentiation, and innate immune response (**Figure 3A**, middle). In the C–B groups, biological processes were enriched in the intrinsic apoptotic signaling pathway in response to oxidative stress and acute-phase response (**Figure 3A**, right). These suggest that DEPs are associated with inflammation and bone formation.

KEGG analysis showed that the DEPs in the B–A groups mainly mediated ribosome, phagosome, glycolysis/gluconeogenesis, complement and coagulation cascades, HIF-1 signaling pathway, etc. (**Figure 3B**, left). Likely, the DEPs in the C–A groups mediated phagosome, glycolysis/gluconeogenesis, pyruvate metabolism, and HIF-1 signaling pathway (**Figure 3B**, middle). The DEPs of the C–B groups mostly participated in endocytosis, ribosome, Huntington disease, prion disease, and Parkinson disease (**Figure 3B**, right).

Then, we used IPA to determine whether the signaling pathways were activated or inhibited with age. The results show that the neuroinflammation signaling pathway, coagulation system, IL-8 signaling, acute-phase response signaling, and CXCR4 signaling are significantly activated in the middle-aged and older groups, suggesting that inflammatory signaling pathways are significantly activated in bone tissue with age (**Figure 3C**). In contrast, EIF2 signaling and glycolysis I, which facilitate bone formation (40–43), are significantly inhibited in the middle-aged and older groups (**Figure 3C**). The complete canonical signaling pathway prediction data are listed in **Supplementary Table 3**.

### Inflammation might be generated from osteocytes

**Figure 4:**
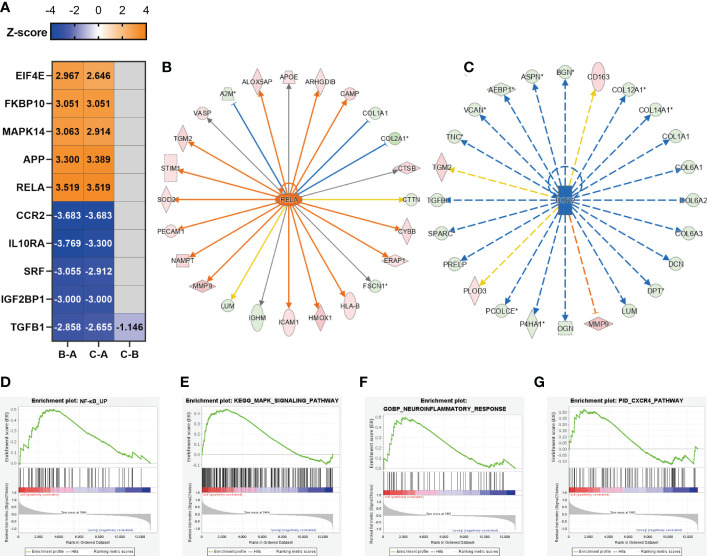
Inflammation might be generated from osteocytes. **(A)** Representative predicted upstream transcription factors in the three comparison groups; the number indicates *z*-score. *Z*-score >2 means the molecular is activated, indicated in orange, while *z*-score <−2 means the molecular is inhibited, indicated in dark blue; the negative prediction was indicated in gray and the non-activated pathway was indicated in light blue. **(B)** The significantly activated molecule RELA and its regulatory map in the C–A groups. **(C)** The significantly inhibited molecule CCR2 and its regulatory map in the C–A groups. **(D–G)** GSEA plots of mRNA sets of several inflammatory-associated signaling pathways.

The upstream regulator analysis (performed by IPA) allowed us to predict transcription factors, small RNAs, and drugs causing the observed protein alterations. The heatmap according to *z*-score shows the top 5 activated and inhibited transcription factors in the three paired comparison groups (**Figure 4A**). The complete upstream regulator prediction data are listed in **Supplementary Table 4**. We identified RELA proto-oncogene, NF-κB subunit (RELA, also known as P65) as the top predicted activated transcription factors of the DEPs between the B–A groups and the C–A groups (**Figure 4A**). As a key subunit of the NF-κB complex, RELA plays an important role in multiple biological processes such as inflammation, immunity, differentiation, cell growth, tumorigenesis, and apoptosis (44). RELA was activated in the bone tissues from middle-aged and older individuals, implying an inflammatory response of bone cells to the aging microenvironment. Upregulation of RELA promotes the expression of CYBB, HMOX1, and ICAM1 which are associated with the neuroinflammation signaling pathway and IL-8 signaling (**Figure 4B**). C-C motif chemokine receptor 2 (CCR2) was the top inhibited transcription factor of the DEPs between the B–A groups and the C–A groups (**Figure 4A**). Downregulation of CCR2 inhibited the expression of bone matrix proteins, such as collagens, BGN, and VCAN (**Figure 4C**), all of which are crucial factors involved in cell adhesion, angiogenesis, and inflammation. In addition, the top 5 activated transcription factors included APP, MAPK14, FKBP10, and EIF4E (**Figure 4A**), of which MAPK14 is an important molecule in the MAPK signaling pathway. The top 5 inhibited transcription factors include IL10RA, SRF, IGF2BP1, and TGFB1 (**Figure 4A**), of which IL10RA is an anti-inflammatory factor (45), while SRF, IGF2BP1, and TGFB1 are all reported to be important molecules in promoting bone formation (46–48).

As described above, the inflammatory response of bone cells was activated along with aging. Since bone cells, including osteoblasts, osteocytes, and osteoclasts, coordinated with each other to maintain bone homeostasis, osteocytes make up over 90% of the cellular content of bone. As the bone samples in which we performed protein sequencing had mainly osteocytes, cellular composition A sequencing data (GSE141595) have shown that osteocytes may be the primary mediator of bone senescence (8). We next explored whether the inflammatory pathways predicted and activated in our study were associated with osteocytes. Previous data showed that RELA and MAPK14 (**Figure 4A**) were predicted to be significantly activated upstream transcription factors, so we focused on whether their corresponding NF-κB signaling pathway and MAPK signaling pathway were activated, which are related to inflammation (44, 49). In addition, the neuroinflammation signaling pathway and CXCR4 signaling were predicted to be significantly activated inflammatory pathways (**Figure 3C**), so we focused on whether the above four signaling pathways were activated. We performed GSEA analysis of published data on osteocyte-enriched tissues (8) (**Figures 4D–G**), and osteocytes in the aged group were enriched in the NF-κB signaling pathway [normalized enrichment score (NES) = 1.5, *p*-value = 0.007], MAPK signaling pathway (NES = 1.49, *p*-value=0.001), neuroinflammatory response (NES = 1.31, *p*-value = 0.11), and CXCR4 pathway (NES = 1.16, *p*-value = 0.23), suggesting that the inflammatory-associated signaling pathways during bone aging were likely to be generated from osteocytes.

### Analysis of the DEPs continuously up- or downregulated with age

**Figure 5:**
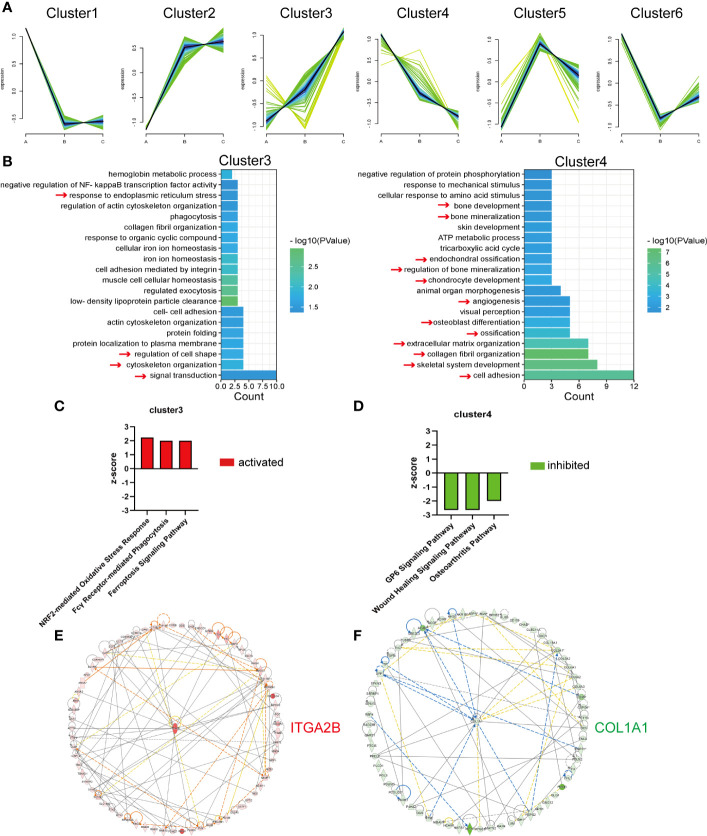
Analysis of the DEPs continuously up- or downregulated with age. **(A)** Six clusters of the 513 DEPs in the three groups. A refers to the children, B refers to the middle-aged individuals, and C refers to the older individuals. **(B)** Representative biological process analysis of cluster 3 (left) and cluster 4 (right). **(C, D)** Activation or inhibition of several canonical signaling pathways in cluster 3 and cluster 4. **(E, F)** The core molecular of cluster 3 and cluster 4.

Chronological expression analysis was applied to better explore protein temporal changes with age. As the C–A groups had the largest age gap, the 513 DEPs (**Figure 2D**) were targeted, and the expression values of these proteins in children, middle-aged individuals, and older individuals were analyzed. K-means clustering analysis was performed on the 513 DEPs, and they were classified into six types based on expression patterns (**Figure 5A**). The number of proteins in cluster 1 to cluster 6 is 93, 96, 70, 65, 72, and 117, respectively. The expression values of the 513 DEPs and proteins of the six clusters are listed in **Supplementary Table 5**. Among the six clusters, DEPs of cluster 3 and cluster 4 were of primary interest to us due to the DEPs upregulated or downregulated continuously with age. The continuously increased or decreased DEPs were subjected to GO-BP enrichment analysis (**Figure 5B**). The continuously upregulated DEPs (cluster 3) were largely involved in signal transduction, cytoskeleton organization, regulation of cell shape, and response to endoplasmic reticulum stress, whereas the continuously downregulated DEPs (cluster 4) were enriched in cell adhesion, skeletal system development, and collagen fibril organization; actually, the overall pathways enriched by continuously downregulated DEPs were closely related with osteogenesis, ossification, and bone mineralization, reflecting that decreased bone formation was a key feature of bone aging (**Figure 5B**).

Then, the IPA program was used to predict the activation/inhibition of the signaling pathways of the continuously up- or downregulated DEPs. We identified three pathways that were significantly activated in continuously upregulated DEPs, namely, NRF2-mediated oxidative stress response, Fcγ receptor-mediated phagocytosis, and ferroptosis signaling pathway (**Figure 5C**), whereas three pathways were significantly inhibited in the continuously downregulated DEPs, namely, GP6 signaling pathway, wound healing signaling pathway, and osteoarthritis pathway (**Figure 5D**).

Moreover, the core molecules in the clusters of continuously up- or downregulated DEPs were selected by the IPA program. Integrin Subunit Alpha 2b (ITGA2B), which increased more than 10-fold (**Supplementary Table 5**) in the older group compared with the children group, was the core molecule among the continuously upregulated DEPs (**Figure 5E**). Collagen Type I Alpha 1 Chain (COL1A1), as the most important bone matrix protein, was the core molecule of continuously downregulated DEPs (**Figure 5F**). COL1A1 was decreased by more than 50% (**Supplementary Table 5**) in the older group compared with the children group, indicating that the reduction of COL1A1 might be primarily responsible for bone aging or aging-related bone loss.

### Potential drug prediction

**Figure 6:**
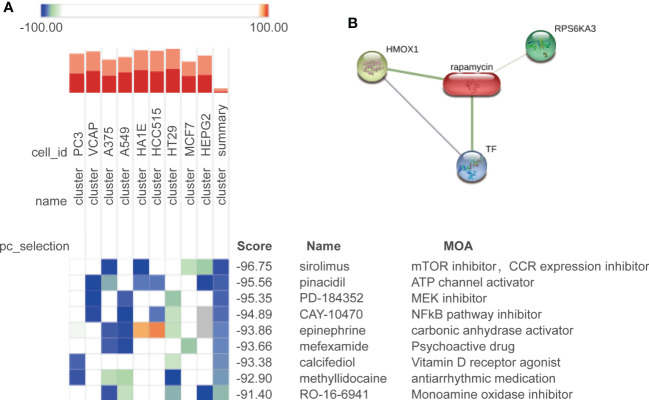
Potential drug prediction. **(A)** Drug candidates predicted by CMap in nine cell types; colors of the heatmap represent the prediction scores: blue means negatively correlated to input genes, and orange means positively correlated to input genes. We show the drugs with a composite score of less than −90. **(B)** Correlation between the predicted drug rapamycin and its target.

To find the potential small molecule drugs against age-related bone loss, we employed the CMap approach to analyze the continuously upregulated (cluster 3) and downregulated (cluster 4) DEPs among the three groups. A total of nine drugs were predicted to be potentially effective (score <−90) (**Figure 6A**). The top predicted drug was sirolimus (also known as rapamycin), and rapamycin forms a complex with FKBP12 and then specifically binds to mTORC1 and inhibits its kinase activity (50). Our prediction suggested a beneficial role of rapamycin against bone cell aging, which was consistent with the current reports characterizing rapamycin as a star drug against cellular aging (50–52). We further analyzed the interaction between rapamycin and the DEPs by using the Search Tool for Interactions of Chemicals (STITCH) database (53). The results showed that rapamycin could interact with HMOX1 (upregulated with age), RPS6KA3 (upregulated with age), and TF (downregulated with age) (**Figure 6B**). Rapamycin also can ameliorate inflammation induced by various stimuli (54–56), which was proper for aged bone in which the inflammatory response was activated in our study.

### Validation of our bioinformatic predictions by *in-vivo* and *in-vitro* experiments

**Figure 7:**
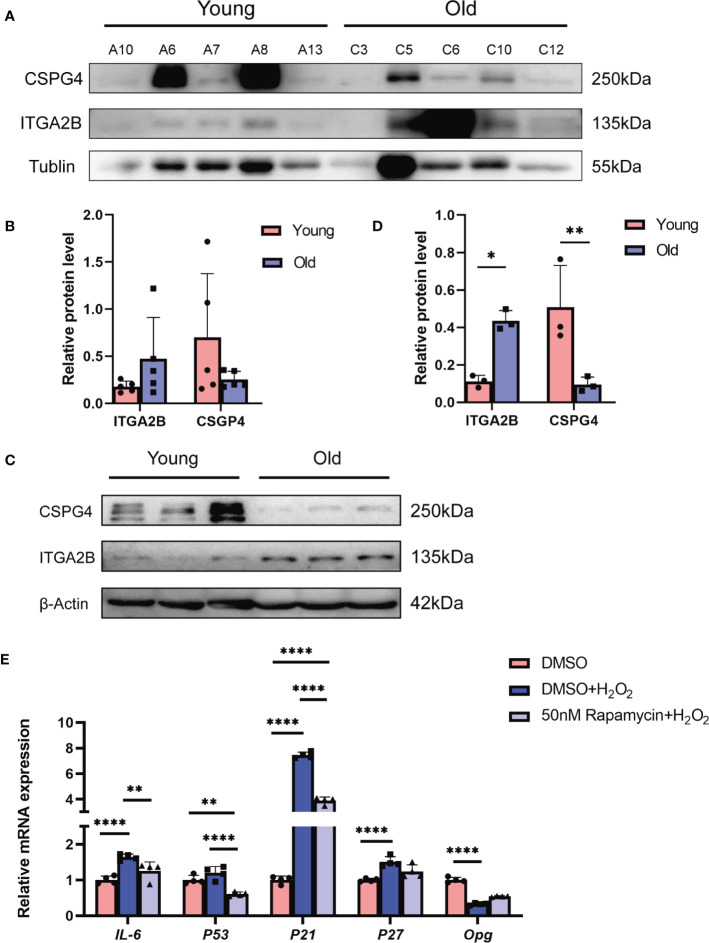
The validation of our bioinformatic predictions by *in-vivo* and *in-vitro* experiments. **(A)** Validation of representative proteins from sequencing. β-Tubulin was used as the control. **(B)** The quantitative results of Western blotting from **(A)**. **(C)** Validation of proteins in young (6 weeks) and old (18 months) mice bone. **(D)** The quantitative results of Western blotting from **(C)**. **(E)** Representative qRT-PCR quantitation for the marker of inflammatory and SASP. All data were presented as the mean ± SD; *p < 0.05; **p < 0.01, **** *p* < 0.0001.

We first validated the expression pattern of the key DEPs from the proteomics sequencing results. The core molecules of the continuously upregulated or downregulated DEPs were ITGA2B and COL1A1, respectively. We observed a severe overexposure of COL1A1 in Western blotting, which may be due to its extremely high abundance in the bone matrix; thus, a cell surface proteoglycan, chondroitin sulfate proteoglycan 4 (CSPG4), another representative downregulated protein, was chosen for further validation. The Western blot assay indicated an increase of ITGA2B and a decrease of CSPG4 in human bone tissues from older individuals than those from children (**Figures 7A, B**), which was consistent with our proteomics sequencing results (**Supplementary Table 5**). The levels of ITGA2B and CSPG4 were also determined in the bone tissues from 6-week-old mice and 18-month-old mice, respectively. In agreement with that of human specimens, ITGA2B was increased significantly, whereas CSPG4 was reduced remarkably in 18-month-old mice (**Figures 7C, D**).

Our bioinformatics analysis showed that the inflammatory-associated DEPs or signaling pathways during bone aging were likely to be generated from osteocytes. As the top-predicted drug against bone aging, rapamycin has been reported to attenuate inflammatory responses. Thus, we explored whether rapamycin reduced the phenotype of cell senescence or senescence-associated inflammation in osteocytes. The mouse osteocyte cell line MLO-Y4 was exposed to hydrogen peroxide (H_2_O_2_) to mimic the senescence microenvironment. The results showed that H_2_O_2_ exposure indeed induced a significant increase of the aging-associated inflammatory cytokine *IL-6* and the senescence markers, including *p53*, *p21*, and *p27*, but there was an obvious decrease of osteoprotegerin (*Opg*), a molecule that inhibits bone resorption; however, rapamycin effectively relieved H_2_O_2_-induced cell damage, indicated by the lower expression of *IL-6*, *p53*, *p21*, and *p27* and the higher level of *Opg* when compared with the H_2_O_2_-treated group (**Figure 7E**). Collectively, we experimentally confirmed the reliability of our proteomics sequencing results and validated the potential of rapamycin against bone aging.

## Discussion

Age-related bone loss remains understudied, and we examined protein changes in the bone tissue of three age groups by proteomics for the first time. In this study, we first characterized the traits of DEPs from pairwise comparison, including DEP numbers and types and GO and KEGG enrichments, respectively. The data indicated that children were markedly different from middle-aged and old individuals with a great number of DEPs and those DEPs were enriched in inflammation and bone formation processes. On this basis, we next analyzed proteins continuously upregulated and downregulated along with age from 513 DEPs screened by comparing old individuals with children. In addition, we predicted drugs that may treat age-related bone loss, with rapamycin as a potential therapeutic agent. In cellular experiments, rapamycin treatment reversed the aging-associated phenotype of MLO-Y4.

Pearson’s correlation analysis of the samples shows that there is a lower intragroup variability in children’s bone tissues, while there is a higher intragroup variability in middle-aged and older individuals’ bone tissues. Although the site of bone tissue collection varied more in children, the sites in middle-aged and elderly people were derived from the hip joint. We speculate that this phenomenon may be due to a combination of factors such as nutritional status, exercise habits, and dietary habits in middle-aged and older adults. Bone tissue samples were obtained from men and women of different ages, and gender was not excluded from the analysis, leading to an overall result that may better describe age-related bone loss rather than postmenopausal osteoporosis. Although we lack direct evidence of bone loss in the elderly samples, the majority of elderly cases were from patients with femoral neck fractures, which could serve as a suggestive basis for bone loss (57–59). It should be pointed out that the reasons for surgery are different in different age groups, and we have tried our best to exclude the influence of systemic factors on bone tissue. However, the influence of bone-related diseases on the local microenvironment cannot be completely excluded. Although the sampling site is far from the lesion, it may still have some influence on the sequencing results.

GO enrichment results suggest that with age, DEPs can be enriched in biological processes associated with inflammation, such as blood coagulation, positive regulation of I-κB kinase/NF-κB signaling, innate immune response, and acute-phase response. We further determined whether inflammatory pathways are indeed activated with age by IPA. The results showed significant activation of various inflammatory signaling pathways, such as neuroinflammation signaling pathway, coagulation system, IL-8 signaling, acute-phase response signaling, and CXCR4 signaling. If the major cells that produce inflammation can be identified, targeting them for intervention may be a way to treat age-related bone loss. We attempted to analyze this by combining single-cell sequencing data, which is currently scarce for bone tissue of different ages, with one study that performed single-cell RNA sequencing of primary human femoral head tissue cells (60). However, their sample size was only four cases, with the younger group being 45 and 31 years old (older than our children group) and already diagnosed with osteoarthritis and osteopenia, obviously not applicable to our study. Considering that the main cell type in the sampling site is the osteocyte, we then selected data from GSE141595, with a tissue source of osteocyte-enriched samples from young and old women, and performed RNA-seq (8), which is closer to our sequencing sample source. The GSEA enrichment analysis reveals that the elderly group is enriched in NF-κB signaling, MAPK signaling, neuroinflammatory response, and CXCR4 signaling. However, IL-8 signaling, acute-phage response signaling, and coagulation system, which were significantly activated in the IPA, were not enriched in the elderly group. It is probably due to that transcriptomics and proteomics are not an exact match, or the difference is caused by the source of the samples which is all women. Although most of the cells in our bone tissue samples are osteocytes, the effects of osteoprogenitors, osteoblasts, and osteoclasts could not be completely excluded.

The current drugs for the treatment of osteoporosis include bisphosphonates, teriparatide, and estrogen, but they are limited by side effects, and research on more effective drugs is necessary. New drugs have been discovered, such as parathyroid hormone-related peptide analogs, sclerostin inhibitors, cathepsin K inhibitors, and senolytics. We selected proteins that consistently increased and decreased with age based on the k-means clustering algorithm and used this to predict potential drugs for age-related bone loss, with rapamycin being the highest-scoring drug. Rapamycin forms a complex with FKBP12 and then specifically binds to mTORC1 and inhibits its kinase activity (50). It has been shown to be an anti-aging drug (51) and has additionally been widely reported as an anti-inflammatory and immunosuppressive agent, but studies of its effects on bone are controversial. Rapamycin alleviated age-related bone trabecular loss in mice (61) and reduced the level of oral inflammation in aged mice (62). Conversely, it has also been reported that rapamycin has a negative effect on bone quality in young mice and rabbit bone tissue (63–65). These results seem to suggest that the effect of rapamycin on bone is dependent on age status. A recent study showed that mTORC1 has age-specific effects on bone (66), which may explain why rapamycin has a two-way effect on bone.

In our study, rapamycin was suggested to attenuate the osteocyte senescence phenotype. We simulated osteocyte senescence by stimulating the mouse osteoid cells MLO-Y4 with H_2_O_2_ *in vitro*. MLO-Y4 produced a significant senescence-related secretory phenotype after H_2_O_2_ stimulation, with significantly elevated mRNA levels of *IL-6*, *P53*, *P21*, and *P27* along with decreased *Opg* levels, while its senescence marker expression decreased and *Opg* levels increased after treatment with rapamycin. Although H_2_O_2_ stimulation is one of the reported methods to induce osteocyte senescence (38, 39), different chemical stimuli or physical radiation does not fully mimic the effects of natural senescence. Although MLO-Y4 is widely used to study osteoblasts *in vitro* (67–70), there are still differences between MLO-Y4 and primary osteocytes; for example, the expression of Sclerostin (*Sost*) is difficult to detect in MLO-Y4 cells (71), which is expressed in primary osteocytes (72). Therefore, it needs to be further validated by primary cells from senescent mice or by animal experiments.

Several other drugs predicted in the CMap database may also be potential drugs for age-related bone loss. The second-ranked drug pinacidil is an oral antihypertensive drug that relaxes vascular smooth muscle and is a K^+^ channel opener (73). Several studies have shown that it prevents damage to osteoblast function from reactive oxygen species and may have a positive effect on bone (74, 75). The third-ranked PD-184352 is a MEK inhibitor, and the MEK/ERK pathway enhances the production of several pro-inflammatory cytokines (76, 77). MAPK14 was predicted to be an upregulated transcription factor in middle-aged and older individuals in our results, and additionally, the GSEA analysis shows that RNA from osteocyte-enriched samples in older women could be significantly enriched in the MAPK pathway, suggesting that targeting the MAPK signaling pathway may be a direction of treatment. It has been shown that PD-184352 inhibits osteoclast differentiation (78), but its effect on osteogenic differentiation is mostly negative (79, 80). In addition, PD-184352 alleviates the phenotype of human rheumatoid arthritis (81), and its study on age-related bone loss was not reported, and further studies are needed in the future. In addition, this study did not target a specific molecule, and the transcription factors predicted by IPA are also the subject of our future research, perhaps to clarify the functions of these transcription factors which might contribute to the discovery of new drugs for age-related bone loss.

In summary, we have utilized proteomics for the first time to characterize age-related bone tissue changes, and based on the proteomics results, we have predicted and experimentally validated potential therapeutic agents, providing a basis for the potential molecular characterization of age-related bone loss.

## Data availability statement

The mass spectrometry proteomics data have been deposited to the ProteomeXchange Consortium (http://proteomecentral.proteomexchange.org) *via* the PRIDE (82) partner repository with dataset identifier PXD039538.

## Ethics statement

The studies involving human participants were reviewed and approved by the Ethics Committee of the Union Hospital of Tongji Medical College, Huazhong University of Science and Technology. Written informed consent to participate in this study was provided by the participants’ legal guardian/next of kin. The animal study was reviewed and approved by the Animal Care and Use Committee of Wuhan Union Hospital.

## Author contributions

ZW, XZ, HW, and JZ contributed to the conception and design of the research. ZW and XZ contributed to the writing and drafting of the manuscript. ZW contributed to the drawing of the figures and tables and analysis of the data. ZW and TR performed the animal experiments. XC, WX, and JL collected the human samples. All authors critically reviewed and approved the manuscript.
