# Supplementary material for: Inflammation produced by senescent osteocytes mediates age-related bone loss
Source: Front Immunol. 2023 Feb 6;14:1114006. doi: 10.3389/fimmu.2023.1114006 (PMC9940315; doi:10.3389/fimmu.2023.1114006)
Supplement: Supplementary file 1 [file Table_1.docx]

| **Group** | **ID** | **Age** | **Gender** | **Diagnosis** | **Sample site** |
| --- | --- | --- | --- | --- | --- |
| A | A1 | 2 | male | old fracture of left external humeral ankle | external humerus ankle |
|  | A2 | 3 | male | left upper ulnar radial joint fusion | ulna |
|  | A3 | 7 | male | left lower limb tibial osteotomy orthopedic | tibia |
|  | A4 | 8 | male | right saddle foot inversion | calcaneus |
|  | A6 | 10 | male | re-fracture after a Monteggia’s fracture | abnormal hyperplastic bone |
|  | A7 | 10 | female | bilateral flat feet | calcaneus |
|  | A8 | 11 | female | bilateral flat feet | calcaneus |
|  | A9 | 11 | female | bilateral flat feet | calcaneus |
|  | A10 | 11 | female | bilateral flat feet | calcaneus |
|  | A12 | 12 | male | humeral defects | ilium |
|  | A13 | 12 | female | unequal length of both lower limbs | ankle joint |
| B | B1 | 41 | female | osteoarthritis, total hip arthroplasty | hip joint |
|  | B2 | 42 | female | osteoarthritis, total hip arthroplasty | hip joint |
|  | B3 | 44 | male | osteoarthritis, total hip arthroplasty | hip joint |
|  | B4 | 45 | male | femoral neck fractures, total hip arthroplasty | hip joint |
|  | B6 | 46 | male | osteoarthritis, total hip arthroplasty | hip joint |
|  | B7 | 50 | male | osteoarthritis, total hip arthroplasty | hip joint |
|  | B8 | 51 | female | osteoarthritis, total hip arthroplasty | hip joint |
|  | B9 | 51 | female | osteoarthritis, total hip arthroplasty | hip joint |
|  | B10 | 53 | male | osteoarthritis, total hip arthroplasty | hip joint |
|  | B11 | 54 | male | osteoarthritis, total hip arthroplasty | hip joint |
|  | B12 | 54 | female | osteoarthritis, total hip arthroplasty | hip joint |
| C | C2 | 88 | female | femoral head necrosis, total hip arthroplasty | hip joint |
|  | C3 | 85 | female | femoral neck fractures, total hip arthroplasty | hip joint |
|  | C4 | 82 | female | femoral neck fractures, total hip arthroplasty | hip joint |
|  | C5 | 81 | male | femoral neck fractures, total hip arthroplasty | hip joint |
|  | C6 | 78 | female | femoral neck fractures, total hip arthroplasty | hip joint |
|  | C7 | 77 | male | osteoarthritis, total hip arthroplasty | hip joint |
|  | C9 | 77 | male | femoral neck fractures, total hip arthroplasty | hip joint |
|  | C10 | 76 | female | femoral neck fractures, total hip arthroplasty | hip joint |
|  | C11 | 74 | female | femoral neck fractures, total hip arthroplasty | hip joint |
|  | C12 | 72 | female | femoral head necrosis, total hip arthroplasty | hip joint |
|  | C13 | 69 | male | femoral head necrosis, total hip arthroplasty | hip joint |

**Table1. The basic information of the 33 individuals included in protein sequencing**
